# Acute Functional Outcomes in Critically Ill COVID-19 Patients

**DOI:** 10.3389/fmed.2020.615997

**Published:** 2021-01-18

**Authors:** Matthew Rong Jie Tay, Poo Lee Ong, Ser Hon Puah, Shuen Loong Tham

**Affiliations:** ^1^Department of Rehabilitation Medicine, Tan Tock Seng Hospital, Singapore, Singapore; ^2^Department of Respiratory and Critical Care Medicine, Tan Tock Seng Hospital, Singapore, Singapore

**Keywords:** SARS-CoV-2, critical illness, coronavirus, acute respiratory distress syndrome, dependent ambulation, intensive care units, dyspnea, muscle weakness

## Abstract

**Background:** COVID-19 (Coronavirus Disease 2019) is a global cause of morbidity and mortality currently. We aim to describe the acute functional outcomes of critically ill coronavirus disease 2019 (COVID-19) patients after transferring out of the intensive care unit (ICU).

**Methods:** 51 consecutive critically ill COVID-19 patients at a national designated center for COVID-19 were included in this exploratory, retrospective observational cohort study from January 1 to May 31, 2020. Demographic and clinical data were collected and analyzed. Functional outcomes were measured primarily with the Functional Ambulation Category (FAC), and divided into 2 categories: dependent ambulators (FAC 0–3) and independent ambulators (FAC 4–5). Multivariate analysis was performed to determine associations.

**Results:** Many patients were dependent ambulators (47.1%) upon transferring out of ICU, although 92.2% regained independent ambulation at discharge. On multivariate analysis, we found that a Charlson Comorbidity Index of 1 or more (odds ratio 14.02, 95% CI 1.15–171.28, *P* = 0.039) and a longer length of ICU stay (odds ratio 1.50, 95% CI 1.04–2.16, *P* = 0.029) were associated with dependent ambulation upon discharge from ICU.

**Conclusions:** Critically ill COVID-19 survivors have a high level of impairment following discharge from ICU. Such patients should be screened for impairment and managed appropriately by rehabilitation professionals, so as to achieve good functional outcomes on discharge.

## Introduction

The Coronavirus Disease 2019 (COVID-19) presents with various degrees of severity, with a significant proportion developing critical illness (1, 2). In these patients, acute respiratory distress syndrome (ARDS) is the most common complication, though encephalopathy, shock, myocardial injury, thromboembolism, and acute kidney injury can also co-exist (3–5). Although it is estimated that many critically ill COVID-19 patients survive (6), it is likely that mechanical ventilation and prolonged bed rest or immobilization can lead to detrimental neuromuscular and cardiorespiratory impairments after critical care, as part of the post-intensive care syndrome (7, 8). Critically ill COVID-19 patients tend to be older and may have comorbid conditions, including hypertension, diabetes mellitus, and cardiac disease (9), which can exacerbate physical function deterioration during critical care.

The surging number of critically ill COVID-19 survivors with widespread disability after discharge from intensive care unit (ICU) is hence expected to present a major rehabilitation need (10). Despite discharge from ICU, these patients may still be functionally dependent due to cardiopulmonary and neuromuscular sequelae of critical illness, such as exertional desaturation (11), platypnea-orthodeoxia (12) and ICU-acquired weakness (ICUAW) (13). Despite the need for acute rehabilitation after ICU, there has been a scarcity of reports on the prevalence and associations of functional dependence in critically ill COVID-19 survivors after ICU stay.

We therefore aim to describe the acute functional outcomes and associations of dependence in walking in critically ill COVID-19 patients after ICU stay. We also describe the cardiopulmonary and neurological sequelae of critical illness contributing to functional dependence.

## Materials and Methods

### Study Design and Participants

We retrospectively reviewed medical records of 62 consecutive critically ill COVID-19 patients admitted to the Intensive Care Unit (ICU) of the National Center for Infectious Diseases, Singapore, between January 1, 2020 to May 31, 2020. The 330-bed National Center for Infectious Diseases, Singapore is the national designated center for COVID-19 patients.

Patients were included if they had laboratory-confirmed COVID-19 and had critical illness as defined by the development of ARDS (14). All patients either required mechanical ventilation or had a fraction of inspired oxygen (FiO_2_) of at least 60% or more (2). All critically ill patients were admitted to the ICU. Laboratory testing for Severe Acute Respiratory Syndrome Coronavirus 2 (SARS-CoV-2) infection was done using SARS-CoV-2 real-time polymerase chain reaction (RT-PCR) of nasopharyngeal, oropharyngeal, or endotracheal aspirate swab samples. There were 2 patients who were admitted to ICU for non-respiratory complications and nine patients who had died during ICU stay. These patients were excluded from this study. The present study was approved by the ethics committee at our institution (NHG DSRB 2020/00639). The requirement for informed consent was waived by the hospital's ethics commission. No sample size calculation was performed due to the exploratory nature of the study. This manuscript adheres to the applicable STROBE guidelines.

### Data Collection

Baseline demographic and clinical characteristics of the patients, including age, sex, ethnicity, body mass index (BMI), premorbid function, comorbidities, and chest radiography findings on admission were extracted from inpatient hospital electronic medical records. In addition to studying individual preadmission comorbidities, comorbidities were also represented using the Charlson Comorbidity Index (CCI), a validated tool for comorbidity adjustment (15). Details of the patient's ICU stay were recorded, including the PaO_2_/FiO_2_ (PF) ratio at admission to ICU, length of ICU stay and the ICU therapies received (high flow nasal cannula, non-invasive ventilation, invasive mechanical ventilation, prone positioning, neuromuscular blockade, extracorporeal membrane oxygenation, vasopressors, and tracheostomy creation). Complications during the ICU stay, namely, ARDS, hospital acquired pneumonia, pneumothorax, myocardial infarction, thromboembolic event, acute renal failure requiring renal replacement therapy (RRT), and the presence of encephalopathy, were also recorded.

### Outcome Measures

The primary functional outcome of patients was measured using the Functional Ambulation Category (FAC) score, which ranges from 0 to 5. This is an observer-assessed score, and we categorized patients into whether they were dependent or independent walkers (16, 17). Dependent walkers required varying degrees of support from another person and were represented by FAC scores of 0 (unable to walk or require 2 or more persons), 1 (requires continuous manual contact), 2 (requires intermittent or continuous light touch) or 3 (requires standby guarding of one person for safety or verbal cueing). An independent walker was represented by an FAC score of 4 and 5, meaning a person who could walk only on level surface or any surfaces including stairs, respectively. A secondary outcome measured was ADL dependence of patients, as defined by being unable or needing help bathing, dressing, toileting, transferring, or eating (18, 19). Another secondary functional outcome was whether patients required supplemental oxygen at rest to achieve a target oxygen saturation of >90% (20). The FAC score, dependence in ADLs and requirement for supplemental oxygen at rest were obtained prior to ICU admission, on the day of transfer out of ICU and the day of hospital discharge based on medical records.

### Physical Sequelae After Critical Illness Contributing to Functional Impairments

Patients were referred for physical therapy after transferring out from ICU if they had functional impairments. These patients were assessed for respiratory or cardiac symptoms. Their heart rate, blood pressure, SpO_2_ (oxygen saturation by pulse oximetry) and muscle strength of the shoulder, elbow, wrist, hip, knee and ankle using the Medical Research Council scale were recorded. These patients also had continuous SpO_2_ and heart rate monitoring if they had respiratory or cardiac symptoms. The cardiopulmonary and neuromuscular sequelae of these patients undergoing physical therapy were then classified into one or more of the following: Orthostatic hypotension (defined as a drop in systolic blood pressure of at least 20 mmHg and/or diastolic blood pressure of at least 10 mmHg within 3 min of standing) (21), presence of exertional dyspnea, exertional desaturation (defined as reduction of SpO_2_ ≤ 90% or relative reduction of 5% during exercise, lasting for 0.5–5.0 min) (11), platypnea-orthodeoxia syndrome (defined as a orthostatic dyspnea and a drop in >5% SpO_2_ or a PaO_2_ > 4 mmHg) (22) or ICUAW (defined as a summed score of <48 with 12 muscle groups being assessed on the Medical Research Council scale or a mean score of <4 in all testable muscle groups) (23, 24). These physical sequelae of critical illness were then compared between patients receiving invasive mechanical ventilation against those that did not have invasive mechanical ventilation. If the patients were not functionally independent for discharge, they were then transferred to an inpatient rehabilitation facility.

### Statistical Analysis

Descriptive statistics were utilized to illustrate patient demographics and clinical characteristics. There were no missing data. The distribution of categorical variables was compared using chi-square or Fisher's exact test. Variables were subjected to univariate analysis investigating their relationship with the primary outcome of independent ambulation upon transfer out of ICU as defined by a FAC score of 4 or 5. These variables analyzed were age, sex, ethnicity, BMI, comorbidities, CCI of 1 or more (25), hospital acquired pneumonia, pneumothorax, myocardial infarction, thromboembolic event, acute renal failure requiring RRT, encephalopathy, PF ratio, prone position, neuromuscular blockade, extracorporeal membrane oxygenation, vasopressors, tracheostomy creation, and length of stay in ICU. Variables which were significant on univariate analysis (age, chronic kidney disease, CCI of 1 or more, hospital acquired pneumonia, thromboembolic event, acute renal failure requiring RRT, tracheostomy, length of stay in ICU) were then subjected to logistic regression analysis. A *P* < 0.05 was considered statistically significant for a two-tailed test. Statistical analyses were generated using SPSS Version 25.0 (IBM Corp., Armonk, New York, USA).

## Results

There were 51 consecutive patients with critically ill COVID-19 enrolled in the study, with the majority being of male gender and Chinese ethnicity. All patients were premorbidly independent in walking and in basic ADLs, and none required supplementary oxygen at rest. The most common preadmission comorbidities were hypertension (41.2%), diabetes (27.5%) chronic cardiac disease (11.8%) and chronic kidney disease (9.8%). Most of the patients had a CCI of 0 (60.8%). All patients had abnormal chest radiography findings on admission, and all had ARDS, with a mean PF ratio (SD) at admission to ICU of 170.1 (55.9). The most common complications in the ICU apart from ARDS were hospital-acquired pneumonia (27.5%), followed by myocardial infarction (15.7%), thromboembolic events (9.8%), acute renal failure requiring RRT (9.8%), pneumothorax (5.9%), and encephalopathy (3.9%). None of the patients had pre-existing neuromuscular disorders or received corticosteroids during ICU admission, and no patients developed stroke as a complication during their hospital stay (which might have impacted their FAC score). There were 28 patients (54.9%) who required invasive mechanical ventilation, with a mean duration (SD) of invasive mechanical ventilation of 15.6 (16.1) days (Table 1).

**Table 1:** Clinical characteristics of study population.

**Characteristics**	***N* = 51**
Age, years, mean (SD)	56.3 (13.1)
Sex, male/female	37/14
Ethnicity, *n* (%)	
- Chinese	31 (60.8)
- Malay	9 (17.6)
- Indian	3 (5.9)
- Others	8 (15.7)
Body mass index, mean (SD)	27.0 (5.38)
Premorbid independent in walking, *n* (%)	51 (100)
Premorbid independent in basic ADLs, *n* (%)	51 (100)
**Preadmission comorbidities**	
- Hypertension	21 (41.2)
- Diabetes mellitus	14 (27.5)
- Chronic cardiac disease	6 (11.8)
- Chronic kidney disease	5 (9.8)
- Chronic obstructive pulmonary disease	1 (2.0)
- Asthma	1 (2.0)
- Chronic neurological disease or dementia	1 (2.0)
- HIV infection	1 (2.0)
- Liver cirrhosis of any Child-Pugh class	1 (2.0)
Charlson Comorbidity Index, *n* (%)	
−0	31 (60.8)
−1	11 (21.6)
- >1	9 (17.6)
Abnormal chest radiography findings on admission, *n* (%)	51 (100)
PF ratio at admission to ICU, mean (SD)	170.1 (55.9)
Complications during ICU stay, *n* (%)	
- ARDS	51 (100)
- Hospital acquired pneumonia	14 (27.5)
- Pneumothorax	3 (5.9)
- Myocardial infarction	8 (15.7)
- Thromboembolic event	5 (9.8)
- Acute renal failure requiring RRT	5 (9.8)
- Encephalopathy	2 (3.9)
ICU therapy, *n* (%)	
- High flow nasal cannula	23 (45.1)
- Non-invasive mechanical ventilation	0 (0)
- Invasive mechanical ventilation	28 (54.9)
- Prone position	30 (58.8)
- Neuromuscular blockade	18 (35.3)
- Extracorporealmembrane oxygenation	1 (2.0)
- Vasopressors	19 (37.3)
- Corticosteroids	0 (0)
- Tracheostomy	5 (9.8)
Length of stay in ICU, days, mean (SD)	14.3 (16.2)

*Data are mean (SD) or n (%). PF ratio, PaO_2_/FiO_2_ ratio; ICU, intensive care unit; RRT, renal replacement therapy; ARDS, acute respiratory distress syndrome*.

Upon transfer out of ICU, there were 24 patients (47.1%) who were dependent walkers (defined as FAC of 0–3) with 22 patients (43.1%) who were dependent in 1 or more basic ADLs. However, upon discharge, a majority achieved independence in ambulation and basic ADLs (92.2 and 90.2%, respectively). All 41 patients (80.4%) who required continuous supplementary oxygen upon transferring out of ICU did not require supplementary oxygen on discharge (Table 2).

**Table 2:** Functional outcomes of study population.

**Characteristics**	***N* = 51**
**Outcomes after transferring out of ICU**, ***n*** **(%)**	
- Continuous supplementary oxygen required	41 (80.4)
- Dependent in walking	24 (47.1)
- Dependent in 1 or more basic ADLs	22 (43.1)
**Outcomes on discharge**, ***n*** **(%)**	
- Continuous supplementary oxygen required	0 (0)
- Dependent in walking	4 (7.8)
- Dependent in 1 or more basic ADLs	5 (9.8)
Require discharge to inpatient rehabilitation facility, *n* (%)	7 (13.7)
Length of stay in acute medical ward, days, mean (SD)	21.9 (17.1)
Total length of stay, days, mean (SD)	36.2 (31.3)

*Data are mean (SD) or n (%). ICU, intensive care unit, ADL, activities of daily living*.

On univariate analysis, we found that an older age, premorbid comorbidity of chronic kidney disease, a CCI of 1 or more, hospital acquired pneumonia, thromboembolic event, acute renal failure requiring RRT, having undergone a tracheostomy and a longer length of ICU stay were significantly associated with dependence in walking as defined by an FAC score of 0–3. However, on multivariate analysis, only a CCI of 1 or more (odds ratio 21.54, 95% CI 2.92–158.84, *P* = 0.003) and a longer length of ICU stay (odds ratio 1.33, 95% CI 1.06–1.66, *P* = 0.013) were identified as significant factors for dependence in walking (Table 3).

**Table 3:** Associations with dependence in walking (defined by FAC 0–3) upon transfer out of ICU.

	**Univariate analysis**	**Multivariate analysis**		
**Characteristics**	***P-*value**	**Odds ratio**	**95% CI**	***P-*value**
**Patient factors**				
Age, years	0.023	0.994	0.880–1.123	0.929
Sex	0.318	–	–	–
Ethnicity	0.720	–	–	–
Body mass index	0.324	–	–	–
Hypertension	0.183	–	–	–
Diabetes mellitus	0.375	–	–	–
Chronic cardiac disease	0.088	–	–	–
Chronic kidney disease	0.013	0	–	1.000
Chronic obstructive pulmonary disease	1.00	–	–	–
Asthma	1.00	–	–	–
Chronic neurological disease or dementia	1.00	–	–	–
HIV infection	0.471	–	–	–
Liver cirrhosis of any Child-Pugh class	1.00	–	–	–
Charlson Comorbidity Index (1 or more vs. 0)	0.008	21.54	2.92–158.84	0.003
**ICU factors**				
Hospital acquired pneumonia	0.005	1.85	0.145–23.51	0.636
Pneumothorax	0.060	–	–	–
Myocardial infarction	0.088	–	–	–
Thromboembolic event	0.012	0	0	1.000
Acute renal failure requiring RRT	0.012	0	0	1.000
Encephalopathy	0.131	–	–	–
PF ratio	0.277	–	–	–
Prone position	0.227	–	–	
Neuromuscular blockade	0.138	–	–	–
Extracorporeal membrane oxygenation	0.284	–	–	–
Vasopressors	0.076	–	–	–
Tracheostomy	0.013	0	0	1.000
Length of stay in ICU, days	<0.001	1.33	1.06–1.66	0.013

*P < 0.05 was considered statistically significant. ICU, Intensive Care Unit; PF ratio, PaO_2_/FiO_2_ ratio*.

Table 4 describes the cardiopulmonary and neuromuscular sequelae of critical illness faced during physical therapy, comparing patients with invasive and non-invasive mechanical ventilation. Most of these sequelae were in patients who received invasive mechanical ventilation, with two patients (7.1%) experiencing orthostatic hypotension, eight patients (28.6%) experiencing exertional dyspnea, 12 patients (42.9%) experiencing exertional desaturation, four patients (14.3%) experiencing platypnea-orthodeoxia syndrome, and five patients (17.9%) experiencing ICUAW. Fewer complications were present in patients receiving non-invasive mechanical ventilation, although there were no statistical differences between both groups.

**Table 4:** Comparison of physical sequelae of critical illness upon transfer out of ICU between patients receiving non-invasive and invasive mechanical ventilation.

**Characteristics**	**Total (*N* = 51)**	**Non-invasive mechanical ventilation (*N* = 23)**	**Invasive mechanical ventilation (*N* = 28)**	***P-*value**
Orthostatic hypotension, *n* (%)	2 (3.9)	0 (0)	2 (7.1)	0.495
Exertional dyspnea, *n* (%)	10 (19.6)	2 (8.7)	8 (28.6)	0.091
Exertional desaturation, *n* (%)	16 (31.4)	4 (17.4)	12 (42.9)	0.051
Platypnea-orthodeoxia syndrome, *n* (%)	5 (9.8)	1 (4.3)	4 (14.3)	0.362
ICU-acquired weakness, *n* (%)	5 (9.8)	0 (0)	5 (17.9)	0.056

Data are n (%). ICU, intensive care unit. Variables were analyzed with the chi-square or Fisher’s exact test.

## Discussion

This study found that nearly half of our critically ill COVID-19 patients had impairments in physical function after ICU care, which were attributable to cardiopulmonary limitations or neuromuscular weakness. This is unsurprising, given that the duration of ICU stay has been reported to be substantially longer in COVID-19 infection at 2 weeks or longer compared to typical ICU populations (26, 27). Our study, similarly, reported an average ICU stay of 14.3 days. The significant functional impairments faced by critically ill COVID-19 survivors are further reflected in the finding that 13.7% of the study's patients were unsuitable for immediate discharge home and required further rehabilitation at an inpatient rehabilitation facility.

We also found significant physical sequelae of critical illness in our study population during physical therapy, especially in patients receiving invasive mechanical ventilation. The significant number of patients (17.9%) who had ICUAW in our study after receiving invasive mechanical ventilation is consistent with the intensive care literature for ARDS patients (23, 28, 29). ARDS survivors have also been reported to have dyspnea and exertion-related desaturation despite not requiring supplementary oxygen at rest, with similar findings reported in critically ill COVID-19 patients (11). A prospective study also found that patients with severe disease had poorer exercise intolerance reflected in lower 6 min walk distance and a higher incidence of diffusing capacity for carbon monoxide impairment compared to patients without severe disease during the early convalescence phase (30). These findings indicate that rehabilitation providers should have a high degree of suspicion for these neuromuscular and cardiopulmonary complications in critically ill COVID-19 survivors.

A unique cardiopulmonary sequelae in our study was COVID-19 associated platypnea-orthodeoxia syndrome, which has been hypothesized to be secondary to alveolar hypoventilation and microangiopathy resulting in gravitational exacerbation of intra-pulmonary shunt in ARDS (12). Additionally, in our cohort of patients receiving invasive mechanical ventilation, nearly half (42.9%) of the patients had exertional desaturation, although only 8 (28.6%) patients had subjective complaints of exertional dyspnea. We suspect that silent hypoxemia, which have been described in patients suffering from COVID-19 pneumonia, persists after the acute phase, explaining the hypoxemic and dyspneic events experienced by the patients (31). This represents a population of patients which will likely benefit from close monitoring during rehabilitation after recovering from the critical phase of the illness.

Apart from close monitoring of patient-reported symptoms and respiratory rate, we found that continuous pulse oximetry and heart rate monitoring during initial rehabilitation was essential in detecting and monitoring for cardiopulmonary rehabilitative complications in critically ill COVID-19 patients, especially given the high prevalence of exertional desaturation. Successful cardiopulmonary rehabilitation strategies that were employed included stepwise mobilization from bed exercises, pre-emptive increases in supplementary oxygen during physical therapy, and interval training sessions (12). A majority of patients were still able to progress to functional independence with none requiring supplementary oxygen on discharge, highlighting the importance of rehabilitation in facilitating discharge planning.

An increased length of stay in ICU and having one or more co-morbidity on the CCI in critical COVID-19 patients were found to be associated with increased dependency upon transfer out of the ICU. This is in keeping with studies in critically ill patients, where length of stay in ICU and the presence of co-morbidities have also been found to be poor prognostic factors (32, 33); similarly, a longer length of stay has been found to be associated with ICUAW in critically ill COVID-19 patients (13). Post-ICU rehabilitation of critically ill COVID-19 patients, though beneficial, may not be feasible for all patients in a resource-constrained setting superimposed with cross-infection risks (34). We believe our study may be useful to identify a subset of patients, especially those with pre-existing health conditions or have a longer length of ICU stay, who may be more susceptible to critical illness sequelae and hence benefit from targeted rehabilitation screening and intervention (35).

There are several limitations in our study. Firstly, we did not utilize other functional measures of impairment or activity limitation such as the 6 min walk test, Functional Independence Measure, or instrumental ADL assessments due to manpower constraints, which might have led to our study underestimating the degree of functional dependence in this population. Secondly, a larger study population will be required to confirm the findings of this exploratory study. Thirdly, patients who died in ICU were excluded from this study. With emerging evidenced-based management guidelines leading to increased survival rates of critically ill COVID-19 patients, it is possible that functional impairments may become more prevalent in this population (36). Fourthly, we only reported acute functional outcomes after ICU stay and hospital discharge. Survivorship issues with regard to long-term mortality, physical function, cognitive function, psychological outcomes and health-related quality of life is likely to be significantly affected in this population, and further study is urgently needed.

## Conclusions

We report a high prevalence of functional impairment and physical sequelae of critical illness in a cohort of critically ill COVID-19 patients. Targeted rehabilitative assessment and management in these patients are crucial in addressing the physical repercussions of COVID-19 related critical illness. Although larger studies are required to confirm the subtypes of patients who are most likely to benefit from targeted rehabilitative assessment, we believe our findings indicate that an individualized rehabilitative approach is vital in the acute convalescent phase to optimize survivorship after critical COVID-19 illness.

## Data Availability Statement

The raw data supporting the conclusions of this article will be made available by the authors, without undue reservation.

## Ethics Statement

The studies involving human participants were reviewed and approved by the National Healthcare Group Domain Specific Review Board. Written informed consent for participation was not required for this study in accordance with the national legislation and the institutional requirements.

## Author Contributions

MT, PO, and ST: contributed to the conception and design of the manuscript and drafting of the manuscript. MT and PO: acquisition, analysis, and interpretation of data. MT, PO, SP, and SL: performed final manuscript review and editing. All authors contributed to the article and approved the submitted version.

## Conflict of Interest

The authors declare that the research was conducted in the absence of any commercial or financial relationships that could be construed as a potential conflict of interest.
